# Exposure to Pornography and Adolescent Sexual Behavior: Systematic Review

**DOI:** 10.2196/43116

**Published:** 2023-02-28

**Authors:** Pranujan Pathmendra, Michelle Raggatt, Megan SC Lim, Jennifer L Marino, S Rachel Skinner

**Affiliations:** 1 Specialty of Child and Adolescent Health Faculty of Medicine and Health The University of Sydney Westmead Australia; 2 Burnet Institute Melbourne Australia; 3 School of Public Health and Preventative Medicine Monash University Melbourne Australia; 4 Melbourne School of Population and Global Health University of Melbourne Melbourne Australia; 5 Department of Obstetrics and Gynaecology Royal Women’s Hospital and University of Melbourne Parkville Australia; 6 Centre for Adolescent Health Murdoch Children’s Research Institute Parkville Australia; 7 Department of Paediatrics University of Melbourne Parkville Australia; 8 Kids Research, Sydney Children’s Hospitals Network Westmead Australia

**Keywords:** adolescence, teenager, sexual debut, sexual behavior, risky behavior, pornography use, digital media

## Abstract

**Background:**

Formative experiences in adolescence lay the foundation for healthy and pleasurable romantic and sexual relationships. Exposure to pornography may affect these experiences.

**Objective:**

We aimed to synthesize evidence published in the past decade on the relationship between exposure to pornography and sexual behavior (earlier age of first sex [<16 years], condomless sex, past-year multiple partners [>1], lifetime multiple partners [>1], group sex, sexual aggression including forced sex, paid sex, teenage pregnancy, and history of sexually transmitted infection) in adolescents aged between 10 and 19 years.

**Methods:**

We identified 19 eligible studies by searching MEDLINE, PsycINFO, Cochrane, CINAHL, Embase, and Web of Science databases from January 2010 to November 2022.

**Results:**

Out of 8 studies that assessed earlier age of first sex, 5 studies, including 1 longitudinal study, found a statistically significant association with exposure to pornography. Given that most studies were cross-sectional or had substantial limitations, causal inference could not be made. Also, exposure to pornography was not measured consistently. The evidence was conflicting or insufficient to draw any conclusions regarding other outcomes.

**Conclusions:**

More quantitative research is needed to elucidate the association between pornography exposure and sexual behavior, and sex education should adopt evidence-based approaches to minimize the potential harms from pornography.

**Trial Registration:**

PROSPERO International Prospective Register of Systematic Reviews CRD42021227390; https://www.crd.york.ac.uk/prospero/display_record.php?RecordID=227390

## Introduction

Adolescence (ages 10-19 years) is a period of marked physical, cognitive, and psychosocial development [1]. Curiosity and experimentation are common in the context of adolescent psychosexual development [2], and adolescents who experience healthy relationships are more likely to have healthy relationships in their young adulthood [3]. However, some sexual behaviors can increase the risk of sexually transmitted infections or unplanned pregnancy [4]. Examples include earlier age of first sexual experience, condomless sex, or sex with multiple partners [5]. Studies have also suggested that teen dating violence, including sexual aggression in adolescence, is linked to substance abuse problems, depression, and psychosis [6]. Hence, studying the factors associated with these types of sexual behaviors in adolescence is crucial for understanding how to best support the healthy development of adolescents.

In adults and older populations of young adults, quantitative studies have demonstrated associations between pornography exposure and behaviors such as sex with multiple partners and condomless sex and sex during intoxication [7,8]. Qualitative and mixed methods studies have also supported this association [9-12]. In the era of en masse digitalization and the widespread use of smartphones and other readily accessible digital media, exposure to pornography has become ubiquitous [13]. Studies in Australia [8] and the United States [14] suggest that adolescents are exposed to pornography by the age of 13 years on average for males and 17 years for females despite laws prohibiting showing or supplying pornography for those aged <18 years [15-18]. This raises concerns as to whether exposure to pornography influences sexual behavior during adolescence.

As noted in a review by Peter and Valkenburg [19] in 2016, there has been increasing empirical research on this subject area. However, whether pornography exposure is causally associated with sexual behavior in adolescence remains contentious [19-23]. It has been argued that adolescents are aware of the shortcomings and artificiality of pornography, hence the impact on sexual behavior is limited [9], whereas others have argued the converse, claiming that pornography exposure is a strong predictor of earlier age of first sex, sexual aggression, and other sexual behaviors [24], as well as a platform upon which adolescents develop their sexual identities and relationships [25]. Previous systematic reviews of the evidence focused on college students [24] and adolescents diagnosed with porn addiction [26] and did not consider some potentially important sexual behaviors such as paid sex [19]. We sought to update the Peter and Valkenburg [19] review in the era of ubiquitous smartphones by systematically reviewing the empirical literature measuring exposure to pornography and its associations with sexual behaviors in adolescents in the general population.

## Methods

### Overview

**Figure 1:**
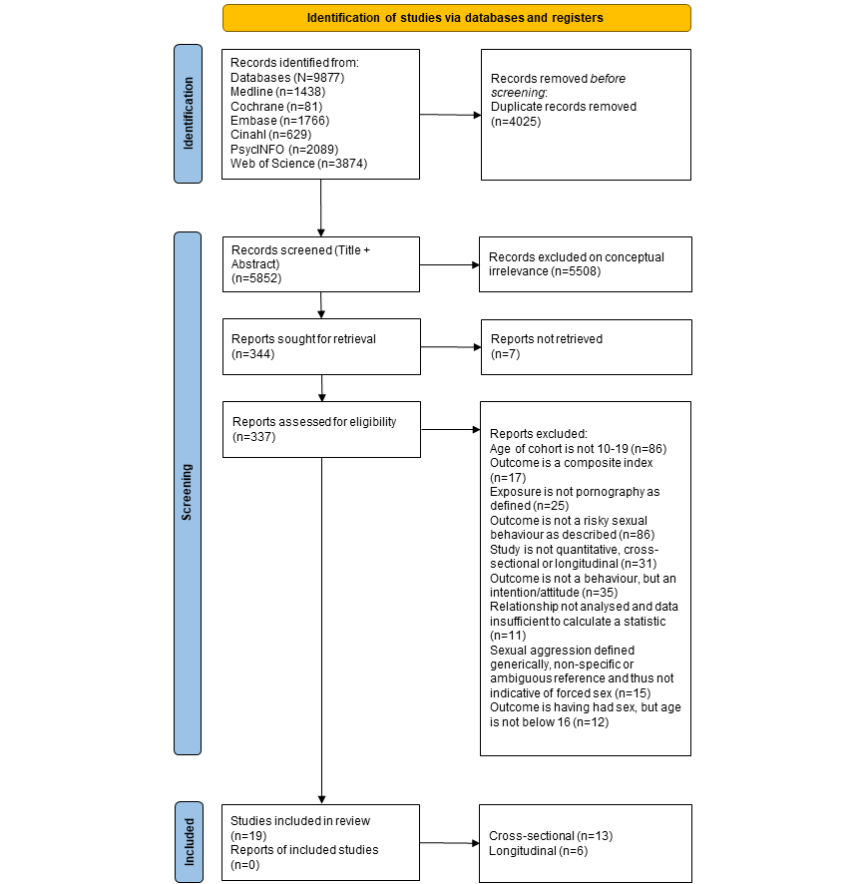
PRISMA (Preferred Reporting Items for Systematic Reviews and Meta-Analyses) flowchart of systematic literature search on adolescent (aged 10-19 years) use of pornography.

We conducted a systematic review of quantitative studies that examined the associations between exposure to pornography and sexual behaviors in adolescents aged between 10 and 19 years. We followed the PRISMA (Preferred Reporting Items for Systematic Reviews and Meta-Analyses) guidelines (Figure 1 and Multimedia Appendices 1 and 2) [27,28]. This systematic review was registered with the International Prospective Register of Systematic Reviews (PROSPERO registration number: CRD42021227390).

We searched 6 databases: CINAHL (via EBSCOhost), Cochrane Reviews (via Ovid, see Multimedia Appendix 3 for specific Cochrane databases searched), Embase (via Ovid), MEDLINE (via Ovid), PsycINFO (via Ovid), and Web of Science (all databases: Web of Science, Current Contents Connect, BIOSIS Previews, CAB Abstracts, and MEDLINE). The search strategy, developed in MEDLINE and later adapted to other databases, used controlled vocabulary and free-text terms that related to the 2 key concepts of adolescents and pornography (Multimedia Appendix 3) as appropriate for each database. The search strategy, including adaptations, was developed by an experienced specialist research librarian. By design, search terms did not include “preteen*” and “prepubert*” because these terms were considered less likely to yield studies about young users of pornography than about child pornography (see *Inclusion and Exclusion Criteria*). Similarly, the names of apps were not included as search terms, because the apps are used not only for pornography by our definition (in subsequent section) but also for interactive sexual experiences. The searches were limited to peer-reviewed papers published from January 1, 2010, to November 30, 2022, to update a previous review article [19]. Other studies that were found to be independent of our search strategy were identified through citation searches.

### Inclusion and Exclusion Criteria

To be considered eligible, studies must have reported quantitative empirical findings on the association between exposure to pornography and sexual behavior outcomes (Table 1). Qualitative studies and systematic reviews were excluded from analysis. For this review, exposure to pornography was defined as a participant’s report of any contact with sexually explicit or erotic media (pornography), with the intent of sexual arousal [29]. We included studies that did not define the exposure but used synonymous terms such as “sexually explicit material,” “adult or X-rated material,” or “pornography” without defining it in the methods. We excluded studies on child pornography, that is, sexually explicit or erotic material portraying individuals aged <18 years.

Sexual behavioral outcomes included those with established associations with adverse health and social outcomes (Table 1). Studies describing sexual aggression were limited to those that measured forced or coercive penetrative sex, excluding those that reported only on forced or coercive kissing and touching or used only the term “sexual acts” without further specification. Composite indices of sexual behaviors were excluded, as the association could not be clearly mapped to a specific behavior. The included studies were limited to those with adolescent participants (aged 10-19 years, inclusive), in which the age range was not provided, and the reported mean or median age was within this range. Included studies were those from a general population sample, and studies with clinical or specific population groups (eg, juvenile detention) were excluded. There was no restriction on the ethnicity of the participants or the study country. Multiple studies that analyzed the same cohort were considered together if they reported different associations.

**Table 1:** List of sexual behavior outcomes considered eligible for present systematic review.

Outcome	Definition
Earlier age of first sex	Reported having had sex under the age of 16 years Reported having had first sex under the age of 16 years Reported mean age of population is below 16 years and participants reported having had sex
Sex for payment OR paid sex	Reported sex with sex workers Reported having paid or received payment for sex
Other sexual behaviors	Reported having had condomless sex Sex with multiple partners or group sex (>1) Multiple sexual partners over lifetime (>1)
Adverse sexual health consequences	Teenage pregnancy Reported history of STI^a^
Sexual aggression OR violence indicative of forced sex (described scale must include forced sex)	Reported perpetration of forced sex (defined or indicative of penetration) Reported being a survivor of forced sex (defined or indicative of penetration)

^a^STI: sexually transmitted infection.

### Data Extraction

The first author (PP) conducted the search and removed duplicates using EndNote X9 [30]. PP reviewed the titles and abstracts (N=5852) and reviewed the full text (n=337) for eligibility (Figure 1). In cases of doubt (n=7), another author (SRS) was consulted. A total of 19 studies were eligible for inclusion. Information about the study location, design, sample size calculation, participants, setting, exposures, outcomes, control variables, methods of statistical analysis, effect sizes, and their variations were extracted by PP and MR. Where possible, effect sizes were calculated from the data if not reported through the study. Included studies either reported on sex or gender. For this systematic review, we used male/female/nonbinary gender or male/female sex to describe the gender or sex of the cohorts as reported in each study.

### Quality Assessment

Study quality was assessed independently by the authors PP and MR using the Newcastle-Ottawa Scale [31]. For the Newcastle-Ottawa scale used to assess longitudinal studies, we replaced the item “was follow-up long enough for outcome to occur” with “whether the outcome was assessed to be at low levels at baseline.” Cross-sectional studies were assessed based on the representativeness of the sample, sample size, addressing the influence of nonrespondents on the reported effect estimates, exposure, and outcome, reporting of statistical analyses, and comparison of outcome and control groups (Multimedia Appendix 4 [32-50]). Longitudinal studies were assessed based on the representativeness of the sample, selection of a nonexposed cohort, assessment of exposure, outcome (at baseline and subsequent waves), follow-up rates, and comparability of outcome and control groups (Multimedia Appendix 4). In cases of conflicts, 2 other authors (ML and JM) were consulted. Low risk of bias was classified as a score of ≥7, moderate risk of bias as a score of 4 to 6, and high risk of bias as a score of 0 to 3.

## Results

### Included Studies

Initial searches identified 5852 abstracts, and 19 studies met the inclusion criteria (Figure 1). This included 11 cross-sectional studies [32-42], 2 case-control studies [43,44], and 6 prospective cohort studies [45-50]. Furthermore, 8 studies addressed early age (<16 years) of first sex [32,34,35,40,41,43,48,50]; 5 studies addressed sexual behaviors including condomless sex [37,42,46,47,49]; 2 studies examined multiple sexual partners during lifetime; a single study examined group sex [35]; 3 studies examined coercive or forced sex [33,39,41]; and 5 studies examined other behaviors including paid sex [38,41,45], teenage pregnancy [44], and history of sexually transmitted infections [36].

The included studies were conducted across 13 countries (Multimedia Appendix 4), and 2 studies were conducted internationally across various countries in Europe [33,40] (Multimedia Appendix 4). Most studies were conducted in European countries (n=9) [33,35,40,41,45,46,48-50], followed by countries in Asia (n=6) [32,36,38,43,44,47], the United States (n=3) [37,39,42], and Eastern Uganda (n=1) [34]. Ten studies reported on the frequency of pornography exposure [33,35,37,38,41,45,46,48-50], and of these studies, one [33] reported “regular” exposure without further definition. Four studies reported exposure to different pornography contents [35,37,39,42], including violent pornography (aggressive sexual acts) and pornography with condomless sex (Multimedia Appendix 4). Exposure to pornography was not uniformly measured; 7 studies did not define pornography in their methods [32,33,35,41,43-45] and a further 6 studies defined it as “sexually explicit content,” “adult content,” “banned media,” or “R-rated media” [34,36-38,40,47] (Multimedia Appendix 4). Similarly, the definitions of outcomes varied across studies. Age at first sex was measured prospectively in one study by asking at each follow-up whether an adolescent had ever had sexual intercourse [50] and retrospectively in 2 studies by asking the age at first sexual intercourse [35,41]. Another longitudinal study assessed the outcome of the first sexual experience in a sexually naive population at baseline, with a mean age at baseline of <16 years [48]. Four studies had a mean sample age of <16 years, so any participant in these samples who had sexual intercourse was considered to have had a young age of first sex [32,34,40,43]. “Multiple sexual partners” included an adolescent self-reporting sex with more than one partner over their lifetime (n=2) [46,47] and having ever participated in group sex (n=1) [35]. Studies of forced or coercive sex included participants as perpetrators (n=3) [33,39,41] and survivors (n=2) [33,39]. Studies of paid sex included participants as buyers (n=3) [38,41,45] or sellers (n=2) [41,45]. Because of the heterogeneity in how exposure and outcomes were measured across studies, a meta-analysis was not possible.

### Early Age of First Sex

A total of 8 studies (2 prospective cohorts, 1 case-control, and 5 cross-sectional) assessed the relationship between pornography exposure in adolescence and earlier age of first sex (defined as below the age of 16 years; Table 2 [32,34,35,40,41,43,48,50]). Of the 6 cross-sectional studies that examined this outcome, 2 studies [35,41] assessed only male adolescents, whereas the other 4 studies [32,34,40,43] studied cohorts of both males and females and the analysis was not stratified by sex. Two prospective cohort studies reported conflicting findings regarding this association. The first study, of 639 male adolescents, conducted in Belgium [50] found a positive longitudinal relationship between exposure to pornography and an earlier age of first sex. The second study [48] reported no association between exposure to pornography and an earlier age of first sex in the overall sample. However, adolescents with a later age of first exposure to pornography had a significantly lower probability of reporting first sexual experience in subsequent waves than their counterparts with earlier exposure to porn. Subsequent sensitivity analyses to account for missing values showed no major association between the timing of porn exposure and first sex in males but showed an association between regular exposure to porn versus no exposure and first sex in females [48]. Both studies were assessed to have a low risk of bias. Four cross-sectional studies of low to moderate risk of bias conducted in Sweden [35], Thailand [32], Eastern Uganda [34], and 23 EU countries [40] supported this association. Two studies did not identify any substantial associations. In 2013, Sahay et al [43] presented a *P* value close to the threshold and conjectured that the rarity of the outcome in their sample limited the power to detect a true association.

**Table 2:** Reported associations between pornography exposure and early age of first sex.

Study (year)				Country of study		Sample size, n (% F/M/NB^a^, gender or F/M sex as reported) and age for analyzed sample, mean (SD)		Risk of bias		Effect estimate, *P* value		Comment on association
**Outcome is reporting the age of first sex below 16 years**												
	**Cross sectional studies**											
		Donevan et al [35] (2017)	Sweden		370 (100% male); NR^b^ (only analyzed 18-year-old males)		Low		Mean age (at first vaginal sex) for frequent viewers is 14.50 years vs average users is 15.61 years vs infrequent users is 15.25 years (*P*=.001). Overall mean age at first vaginal sex is 15.42 years		Frequent users were more likely to try out sexual acts seen in pornography (*P*=.002) Mean age at first anal sex is >16 years	
		Svedin et al [41] (2011)	Sweden		Reference group of nonfrequent viewers of pornography, 13.5% of n=1429-1702 (100% male) Frequent users of pornography, 19.5% of n=172-200 (100% male) Overall, 18.15 (0.74) years		Low		AOR^c^ 0.77, 95% CI 0.48-1.23; *P*=.27		Frequent users were also more likely to report believing porn to be one of the best ways to learn about sex (38.1% vs 18.1%; *P*<.001) Adjusted for other covariates including background sociodemographic status, parental relationship, problems in conduct, and other sexual behaviors Significant when nonadjusted	
**Outcome is reporting of any sex, but mean age of cohort is below 16 years**												
	**Cross-sectional study**											
		Atwood et al [32] (2012)	Thailand		420 (50.5% female) 13.45 (0.5) years		Moderate		AOR 2.73, 95% CI 1.25-5.96; *P*<.01		Adjusted for parental relationships, relationship status, sex refusal, and self-efficacy	
		Bukenya [34] (2020)	Uganda		598 (52% male) 14.2 (2.6) years		Low		AOR 2.29, 95% CI 1.60-3.29; *P*=NR		46.3% of sample was aged 15-19 years Adjusted for covariates including age, education level, parental relationship, substance use, sexing experience, history of being bullied, experienced physical arrack, intentions of participating in verbal sex jokes, and engagement in labor activities	
		Sahay [43] (2013)	India		205 (47% female) 14.6 (NR) years		Moderate		AOR 2.6, 95% CI 0.95-7.05; *P*=.06		Low proportion of population reporting outcome; 31.7% (13/41) of case defined as having ever had an intimate relationship, and 28.6% (44/164) of control were aged 16-19 years Adjusted for medium of instruction, access to sexual health material, history of sexual abuse, parental relationship, and reported STD^d^ symptoms	
		Ševčíková et al [40] (2014)	EU countries		11,712 (50% female) Overall mean age NR, but across categories of porn exposure, mean age ranged from 13.24 to 14.12 years		Low		AOR 3.25; β^e^=1.18, SE 0.12; *P*<.01		Adjusted for restrictive parental mediation, gender, and national location (indicative of liberalism in wider context); model particularly adjusted for these variables, not covariates	
	**Prospective cohort study**											
		**Matković et al [48] (2018)**										
			Croatia (Rijeka)		866 (39% male) At baseline, n=1037, 15.8 (0.5) years		Low		Moderately frequent exposure Male: β=0.89, SE 0.59; *P*=.128 Female: β=0.09, SE 0.29; *P*=.754 Most frequent exposure Male: β=0.14, SE 0.69; *P*=.837 Female: β=0.76, SE0.39; *P*=.051 Age of exposure to porn, males: β=−1.72 to −1.79; *P*<.05		Adjusted for pubertal status, education (school type), age at first exposure to porn, age, parental mediation, sexual intention (in the form of sensation seeking), and a contextual variable indicative of peer participation in sex. Compared with males with earliest exposure to porn, those who had later exposure to porn had significantly lower probability of reporting the outcome. Nonsignificant in females	
			Croatia (Zagreb)		n=793 At baseline, 16.1 (0.44) years, and 67.8% female				β=0.63; *P*<.05		Replication analysis, associations in Rijeka panel were not corroborated Analysis is comparing regular users of porn to those who do not use porn	
		Vandenbosch et al [50] (2013)	Belgium		584 (58% male in overall population of n=639) 14.78 (1.18) years at baseline, study is in 2 waves, 6 months apart		Low		AOR 4.96, 95% CI 1.34-18.40; *P*=.02		Comparing frequent users of pornography to nonfrequent users Adjusted for variables by including control variables such as country of origin, gender, age, educational level, parental relationships, peer communication, and sensation seeking, which were added as predictors in analyses at wave 2	

^a^F/M/NB: female/male/nonbinary.

^b^NR: not reported.

^c^AOR: adjusted odds ratio.

^d^STD: sexually transmitted disease.

^e^β: unstandardized regression coefficient.

### Condomless Sex

A total of 5 studies (3 prospective cohorts, 1 case-control, and 1 cross-sectional) assessed the relationship between pornography exposure in adolescence and participation in condomless sex (Table 3 [37,42,46,47,49]). Two studies [37,42] were conducted in the United States, and one each in Croatia [46], Taiwan [47] and the Netherlands [49]. The largest study assessing this outcome was a prospective cohort study of 2054 participants conducted in Taiwan [47], and the smallest was a cross-sectional study of 206 participants conducted in the United States [37]. Of the 2 cross-sectional studies that examined this outcome, one studied a cohort of male adolescents [37], whereas the other examined a cohort of males and females, where analysis was not stratified by gender [42]. The 3 longitudinal studies reported cohorts of male and female adolescents, where the analysis was not stratified by sex. However, one study [46] included gender as an interaction term to analyze the effects of gender on the association, while another [47] adjusted for gender in the regression models.

The results of these studies were mixed. Among 3 longitudinal studies [46,47,49], one conducted in Taiwan [47] reported a positive association and was assessed to have a moderate risk of bias, while the studies from Croatia and the Netherlands [46,49] reported no major associations and were assessed to have a low risk of bias. Both cross-sectional studies supported a positive association between exposure to pornography and condomless sex in the samples [37,42]. Nelson et al (2019) examined pornography patterns in non-heterosexual male adolescents [40], a study that was assessed to have a low risk of bias. Specifically, this study found that those who viewed more condomless sex (>50% of total pornography viewed) were more likely to have reported participating in condomless sex [37]. Another cross-sectional study that found an association, by Wright et al [42], was assessed to have a high risk of bias owing to poor adjustment of confounders and ascertainment of exposures and outcomes.

**Table 3:** Reported associations between pornography exposure and sexual behaviors.

Study (year)			Country of study	Sample size, n (% F/M/NB^a^, gender or F/M sex as reported) and age for analyzed sample, mean (SD)	Risk of bias	Effect estimate, *P* value	Comment on association
**Condomless sex**							
	**Cross-sectional studies**						
		Nelson et al [37] (2019)	United States	206 (100% male) 16 (1.0) years	Low	AOR^b^ 2.4, 95% CI 1.1-5.2; *P*=NR^c^	Referred comparator group is those who reported <50% of porn viewed contains condomless anal sex Adjusted for age and ethnicity
		Wright [42] (2020)	United States	n=95 adolescents who reported having had intercourse in the last year (56.6% female) 45.4% of case are aged 18 years	High	OR^d^ 1.92, 95% CI 1.23-2.98; *P*=NR	Odds ratio increased to 2.97 (1.48-5.93) when parents did not discuss any topics about sexual health Parental communication about sex was used as a conditional variable
	**Prospective cohort studies**						
		Koletić et al [46] (2019)	Croatia	Zagreb cohort: at baseline: n=1057, (35.6% male), 16.14 (0.45) years; analyzed sample in model, n=246 Rijeka cohort: at baseline: n=1071 (38.4% male), 15.82 (0.49) years; analyzed sample in model, n=297	Low	Zagreb cohort: AOR 1.02, 95% CI 0.84-1.24; *P*>.05 Rijeka cohort: AOR 0.99, 95% CI 0.78-1.28; *P*>.05	Association was nonsignificant in nonadjusted model; potential correlation between initial baseline exposure to porn and condomless sex at final time point not examined Adjusted for parents’ education, age, pubertal timing, sensation seeking, and multiple partners; interaction term of gender and pornography use was found to be nonsignificant
		Lin et al [47] (2020)	Taiwan	N=2054 (51% male at baseline, n=2690) At baseline, 13.3 (0.49) years	Moderate	β^e^=0.274, SE 0.131; *P*<.05	Adjusted for gender, parental education level, socioeconomic status, socioeconomic status, status of family (including cohesion, number of siblings), education level, relationship status, history of depressive symptoms, and impact of participant’s school
		Peter et al [49] (2011)	The Netherlands	N=1445 (51% male); at baseline, 14.49 (1.68) years	Low	AOR 0.980, 95% CI 0.658-1.459; *P*=NS^f^	Association was significant in the adult cohort; when age interaction terms were included in logistic regression models, nonsignificant associations were observed across both cohorts Controlled for sensation seeking, life satisfaction, peer relationships, sexual orientation, relationship status, number of lifetime sex partners, and casual condomless sexual behavior of friends
**Group sex**							
	**Cross-sectional studies**						
		Donevan and Mattebo [35] (2017)	Sweden	370 (100% male), NR (18-year-olds); adolescents who reported having had group sex (n=23)	Low	OR 1.65, 95% CI .48-5.73; *P*>.05	Odds ratio calculated by this review’s authors Frequent and average users of porn as reported by study were used as the exposed group; infrequent users were used as the control group
**Lifetime sexual partners**							
	**Prospective cohort studies**						
		Koletić et al [46] (2019)	Croatia	Zagreb cohort: at baseline: n=1057, (35.6% male), 16.14 (0.45) years; analyzed sample in model, n=246 Rijeka cohort: at baseline: n=1071 (38.4% male), 15.82 (0.49) years; analyzed sample in model, n=293	Low	Zagreb cohort: AOR 1.31, 95% CI 0.94-1.73; *P*>.05 Rijeka cohort: AOR 1.05, 95% CI .77-1.43; *P*>.05	Association was significant in nonadjusted model; potential correlation between initial baseline exposure to porn and condomless sex at final time point not examined Adjusted for parents’ education, age, pubertal timing, sensation seeking, and multiple partners; interaction term of gender and pornography use was found to be nonsignificant Assessed 2 or more sexual partners over lifetime
		Lin, Liu, and Yi [47] (2020)	Taiwan	N=1477 (51% male at baseline, n=2690); at baseline, 13.3 (0.49)	Moderate	β=2.725, SE 1.059; *P*<.05	Positive significant association when exposure considers multiple media (internet, magazines, etc) of porn rather than specific types of pornography Adjusted for gender, parental education level, socioeconomic status, socioeconomic status, status of family (including cohesion, number of siblings), education level, relationship status, history of depressive symptoms, and impact of participant’s school Assessed 2 or more sexual partners over lifetime

^a^F/M/NB: female/male/nonbinary.

^b^AOR: adjusted odds ratio.

^c^NR: not reported.

^d^OR: odds ratio.

^e^β: unstandardized regression coefficient.

^f^NS: nonsignificant *P* value.

### Multiple Sexual Partners

Two prospective studies examined the association between pornography exposure and multiple lifetime sexual partners [46,47]. These studies were conducted in Croatia [46] and Taiwan [47]. Both studies examined male and female cohorts. Koletić et al [46] included gender as an interaction term to analyze the effects of gender on the association, whereas Lin et al [47] adjusted for gender in the regression models. The study by Lin et al, 2020 [47] found an association between pornography exposure and multiple sexual partners and was assessed to have a moderate risk of bias owing to not measuring the outcome at baseline, having a low proportion of the sample reporting the outcome, and limited assessment of follow-up and retention rates (Table 3). This study reported exposure to pornography across different media (such as comic books and web sites) in a single exposure variable. Koletić et al [46] found a major association but only in a nonadjusted model. A single cross-sectional study conducted in Sweden on a cohort of male adolescents reported a nonsignificant association between exposure to pornography and participation in group sex [35], that is, sex with multiple partners simultaneously (Tables 1 and 3).

### Forced or Coercive Sex

A total of three cross-sectional studies [33,39,41] assessed the relationship between pornography exposure in adolescence and experiences of forced or coercive penetrative sex (Table 4). These studies were conducted in the United States [39], Sweden [41] and 5 European countries [33]. The largest study was by Barter et al [33] in 2021 of 3277 adolescents, and the smallest was conducted by Rostad et al [39] in 2019 with 1766 adolescents. Furthermore, 2 out of 3 studies reported a major positive association in reported experiences of forced or coercive sex in males only [33,39]. Two of the 3 studies [33,39] stratified their analyses as male or female, and one of the studies examined only male adolescents [33].

**Table 4:** Reported associations between pornography exposure and forced/coercive sex, paid sex, teenage pregnancy, and history of sexually transmitted infection.

Study (year)				Country of study		Sample size, n (% F/M/NB^a^, gender or F/M sex as reported) and age for analyzed sample, mean (SD)		Risk of bias		Effect estimate, *P* value		Comment on association
**Forced or coercive sex**												
	**Cross-sectional studies**											
		Rostad et al [39] (2019)	United States		N=1766 responded (52.3% female), 15.42 (0.65) years Analysis performed on: n=746 (female) and n=578 (male)		Low		Male victimization: AOR^b^ 2.60, 95% CI 1.40-4.83 Female victimization: AOR 1.63, 95% CI 0.96-2.76 Male perpetration: AOR 3.34, 95% CI 1.85-6.04 Female perpetration: AOR 0.99, 95% CI 0.39-2.55		Adjusted for age, socioeconomic status, substance use, history of suspension or expulsion from school, tolerance of rape myths, attitudes based on gender, experiences of drinking, and marijuana use Analysis stratified by gender	
		Svedin et al [41] (2011)	Sweden		Overall, 18.15 (0.74) years Analyzed sample: n=172-200 (100% male) adolescents who were frequently exposed to porn; n=1429-1702 (100% male) reference group of male adolescents who were less frequently exposed to porn		Low		AOR 1.49, 95% CI 0.80-2.76; *P*=.18		Adjusted for other covariates including background sociodemographic status, parental relationship, problems in conduct, and other sexual behaviors	
		Barter et al [33] (2021)	England, Italy, Norway, Bulgaria, and Cyprus		N=4564 (NR^c^) NR (14-17) years (14.7 years in England and 15.3 years in Italy) Analysis conducted on n=3277		Low		Male perpetration: AOR 2.46, 95% CI 1.80-3.34		Nonsignificant association in females, not reported Adjusted for age, country of residence, negative gender attitudes, aggression in peers, experiences of bullying, and violence in household	
**Paid sex (bought sex)**												
	**Cross-sectional studies**											
		Ng and Wong [38]^d^ (2016)	Singapore		300 (100% male), median=8 (IQR 18-19) years		Low		AOR 1.47, 95% CI 1.04-2.09		Adjusted for alcohol consumption, rebellious attitudes, self-esteem, perceived external control, academic performance, participation in cocurricular activities, age of first sex, and history of having a sexually active girlfriend	
		Svedin et al [41] (2011)	Sweden		Overall, 18.15 (0.74) years Analyzed sample: n=172-200 (100% male) adolescents who were frequently exposed to porn; n=1429-1702 (100% male) reference group of male adolescents who were less frequently exposed to porn		Low		AOR 1.71, 95% CI .71-4.14; *P*=.23		Adjusted for background sociodemographic status, parental relationship, problems in conduct, early ages of first sex, paid sex, experiences of sexual aggression, and perceived feelings of sexual lust	
	**Prospective cohort studies**											
		Averdijk [45] (2020)	Switzerland		602 (males only) At baseline (n=1675), overall: 13.7 (0.37) years		Moderate		OR^e^ 1.284; β coefficient 0.250; *P*<.01		Stratified by gender, analyzed males only owing to no prevalence of outcome in females	
**Paid sex (sold sex)**												
	**Cross-sectional studies**											
		Svedin et al [41] (2011)	Sweden		Overall, 18.15 (0.74) years Analyzed sample: n=172-200 (100% male) adolescents who were frequently exposed to porn; n=1429-1702 (100% male) reference group of male adolescents who were less frequently exposed to porn		Low		AOR 2.68, 95% CI 1.13-6.35; *P*=.03		Adjusted for background sociodemographic status, parental relationship, problems in conduct, other sexual behaviors, and perceived feelings of sexual lust	
	**Prospective cohort studies**											
		Averdijk [45] (2020)	Switzerland		1197 (52% of target population, n=1675 is male) At baseline: 13.7 (0.37) years		Moderate		AOR 0.463; B coefficient −0.770; *P*<.01		Association is conditional on gender; high levels of porn use amplified the difference (females had a higher level of selling sex than males) Odds ratio was adjusted for gender by including it as an interaction term Unadjusted model was nonsignificant	
**Teenage pregnancy**												
	**Case-control analysis**											
		Siti-HaidahSiti-Haidah et al [44] (2017)	Malaysia		215 (100% female) 75% above 15, age range of 12-19 years		Moderate		AOR 9.9, 95% CI 4.3-22.5; *P*<.001		Adjusted for background demographics including, ethnicity, socioeconomic status, and parental education	
**History of sexually transmitted infections**												
	**Cross-sectional studies**											
		Kim et al [36] (2016)	South Korea		2387 (25.4% female) NR (12-19 years age range)		Low		Male: AOR 2.623, 95% CI 1.214-5.668 Female: AOR 14.00, 95% CI 2.150-91.170		Analysis stratified by gender	

^a^F/M/NB: female/male/nonbinary.

^b^AOR: adjusted odds ratio

^c^NR: not reported.

^d^Reported median age of cohort (IQR).

^e^OR: odds ratio.

### Paid Sex

The relationship between pornography exposure and “paying for sex” was inconsistent (Table 4). Studies have been conducted in Singapore [38], Sweden [41], and Switzerland [45]. A prospective cohort study [45] reporting this exposure and outcome found a positive association between exposure to pornography and sex. This was supported by one [38] of the 2 cross-sectional studies [38,41] that examined this association. There are an insufficient number of studies examining both male and female adolescents to conclude whether this association was gender dependent, as both Ng et al [38] and Svedin et al [41] reported on male adolescents yet found opposing substantial associations. Regarding the outcome of selling sex, a prospective cohort study found no association with pornography viewing [45]. However, they reported an interaction effect with gender, whereby females with high pornography consumption were more likely to sell sex than those with low consumption. One cross-sectional study [41] that sampled only male adolescents found a positive association between selling sex and viewing pornography. There are an insufficient number of studies examining this association to draw conclusions.

### Sexual Health Consequences

One study reported a major association between exposure to pornography and teenage pregnancy [44], whereas another found an association with a history of sexually transmitted diseases [36] (Table 4). The study of teenage pregnancy was assessed to have a moderate risk of bias, largely due to poor assessment of nonrespondents and limited justification of sample size. Both studies were limited by a lack of representative samples.

## Discussion

We identified 19 papers published in the past 10 years that reported the association between pornography exposure and sexual behaviors in adolescents. Our review found that exposure to pornography in adolescence is associated with an earlier age of first sex (below 16 years of age). Notably, recent studies with higher-quality evidence supported the existence of a relationship between exposure to pornography and earlier age of first sex for adolescents aged 10-19 years. Out of the studies, 4 cross-sectional studies [32,34,35,40] and 1 longitudinal study found this association. Two cross-sectional studies did not report a significant association, with the study by Sahay et al [43] limited by a low proportion of the population reporting the outcome, and the study by Svedin et al [41] limited by the retrospective nature of the reported outcomes and the era in which data collection occurred (before access to smartphones and high-resolution internet video). Another longitudinal study by Matković [48] also found inconsistent associations between pornography exposure and age of first sex, depending on how the exposure was measured (timing and quantity) and the gender of the adolescent. However, these were not consistently present in all analyses. The study was limited by the baseline mean age of the sample being 15.8 (SD 0.5) years and the fact that sexual debut soon after this age would be expected. A review by Peter and Valkenburg in 2016 [19] also reported an association between pornography exposure and earlier age of sexual activity, and our review has strengthened this finding with the inclusion of additional, more recent studies. We did not find an association between exposure to pornography and any other sexual behaviors. Notably, a positive association between exposure to pornography and earlier age at first sex in adolescence does not imply a causal relationship, even when a temporal association is identified, as in a longitudinal study [50]. For example, adolescents who are more interested in sexual activity and therefore more likely to become sexually active may also be more likely to view pornography because of their interest in it. Both Donevan et al [35] and Svedin et al [41] reported higher exposure to pornography in those seeking it as a source of inspiration or knowledge about sex.

There was a limited number of studies, mainly cross-sectional, examining the relationship between exposure to pornography and forced sex, paid sex, multiple partners, teenage pregnancy, and history of sexually transmitted infection [33,35-39,41,42,44-47,49]. There is clearly a need for more longitudinal studies to determine whether exposure to pornography is associated with other sexual behaviors in adolescents. In contrast to previous reviews [19,26], we examined forced penetrative sex separately from sexual aggression (aggressive intentions or coerced nonpenetrative sexual behaviors such as kissing or touching). We found mixed results, which might be clarified if the studies were to report these outcomes separately. We excluded some studies [22,51,52] where forced sex was measured only as “forced sexual acts” and hence accounted for forced sexual behaviors that are strictly not forced sexual intercourse, such as kissing or touching against one’s will. It is also worth noting that one study included in this review found an association between exposure to pornography and forced sex [39] focused specifically on violent pornography, defined as any erotic media depicting individuals being forced into sexual acts. A recent meta-analysis found that violent pornography was modestly correlated with sexual aggression and noted difficulties in interpreting the data owing to citation biases and researcher expectancy effects [53]. In addition, a systematic review conducted in 2021 aligned with our observations, noting the difficulty of clarifying the association between pornography consumption and nonconsensual acts such as rape [54]. This may differ from the impact of nonviolent pornography, which has been associated with reduced sexual aggression in older populations [11,53,55]. It is possible that exposure to violent pornography is associated with sexually aggressive behavior.

Before considering the significance of our findings, we recognize that our systematic review was not without its limitations, particularly the potential for missing literature. This may have been the case because our choice of search terms and databases was not sent for external peer review [27,28], and we excluded grey literature. However, using a wide array of databases spanning across multiple disciplines (Multimedia Appendix 3), consulting with an expert librarian, and searching citations of included articles, we conducted a comprehensive search to address the aims of this review. This review included studies from 13 different countries and 2 cross-national European studies (Appendix 4). Most studies were conducted in high-income Western settings. For multiple lifetime sexual partners, no major association with pornography exposure was found when the study was conducted in a Western country (Croatia) [46], but there was an association in an Eastern country (Taiwan) [47]. A similar pattern was identified for the outcomes of group sex, teenage pregnancy, and history of sexually transmitted infections (Tables 3 and 4), yet there are insufficient studies examining or comparing culturally diverse locations to draw conclusions. The role of cultural influence on the relationship between pornography exposure and sexual behaviors is under-studied in adolescents, but this review suggests that country and culture may be important. Several articles assessing the relationship between exposure to pornography and outcomes of sexual behavior in older populations have suggested the significance of cultural context as an influence on how susceptible an individual is to adopt the sexual scripts prescribed in pornography [56-58]. In certain cultures, where access to sexual health education is limited, adolescents tend to use pornography as one of the few sources of sexual health knowledge [59]. It is possible that in societies without sex education, adolescents may more readily adopt sexual scripts presented in pornography [60,61], normalizing sexual behaviors depicted in pornography. Despite this conjecture, the role of culture in adolescents’ exposure to pornography and its relationship with sexual behaviors remains unknown, and further studies are recommended.

Inconsistent findings on the association between exposure to pornography in adolescence and sexual behavior also likely reflect the limitations of research studies. Consistent with a previous review [19], we found that exposure to pornography was not assessed in a uniform or reproducible manner (Multimedia Appendix 4). Nearly half of the studies did not define pornography in their methods, and a further 5 studies defined it simply as “sexually explicit content,” “adult content,” or “banned material.” A lack of specificity in exposure may impact a study’s ability to identify an existing association. It also prevents the pooling of data in a meta-analysis, limiting the ability to increase the power to detect an association. There is a clear need to standardize the definition and quantification of pornography. Two studies identified that adolescents commonly reported using pornography as an information source [35,41]. Such ideas align with previous findings that adolescents learn about sex, sexual identities, and sexuality through pornography [9,62]. Pornography content, perceptions of that content, frequency of use, and reasons for use are important dimensions of pornography exposure. A consistent and reliable measurement of these dimensions will help elucidate the pathways between pornography and sexual behavior in adolescents. In addition, as in the case of studies assessing sexual aggression indicative of forced sex (Table 4 and Multimedia Appendix 4), questionnaires assessing sexual behaviors require careful piloting and validation to ensure that they are understood by adolescents as intended. Furthermore, statistical analyses that do not combine forced sex with other sexually aggressive acts into one outcome can help to further clarify the observed associations. A review of how sexual aggression has been measured and reported thus far in the literature could help clarify the literature on pornography use and its associations with forced sex. To further clarify the nature of the observed associations, studies should also control for baseline confounders, such as access to or the quality of sexual health knowledge received by adolescents. While we recommend further research to develop more reliable and robust measures of exposure and outcomes, we also recognize that this type of research on adolescents’ behavior presents ethical barriers. There is a high level of moral concern in society regarding the potentially harmful influence of pornography on adolescents, including among research participants. This concern is likely to translate into barriers to the approval and funding of research on this topic.

Most studies in this review were not inclusive of adolescents who were sexuality or gender-diverse. It has also been reported that same-sex-attracted male adolescents use pornography to negotiate their sexual identity, learn about the mechanics of sex and performance, and define ideas of pain and pleasure [63]. Another study found that sexuality diverse adolescents used social media to share sexual information [64]. Hence, it is possible that pornography may have different associations with sexual behaviors in sexuality diverse adolescents than in cisgender heterosexual peers. We recommend studies on sexual behaviors and their relationship to pornography in sexuality and gender-diverse adolescents.

Recent findings [65] suggest that sexual attitudes mediate the relationship between pornography exposure and sexual behavior. Recent studies in the United States [66] have found that adults use pornography as a source of sex information. In college students, studies suggest a mediating role of sexual education in the relationship between exposure to pornography and sexual behaviors [67-69]. It is possible that individuals are most strongly influenced by pornography when they have limited access to sex education. Therefore, supporting international guidelines [70], we also recommend that sex education be school based, universal, comprehensive, accurate, evidence informed, and age appropriate.

## Appendix

Multimedia Appendix 1 PRISMA (Preferred Reporting Items for Systematic Reviews and Meta-Analyses) checklist (2020) for systematic review on adolescent (aged 10-19 years) use of pornography.

Multimedia Appendix 2 PRISMA-S (Preferred Reporting Items for Systematic Reviews and Meta-Analyses Literature Search Extension) checklist for systematic review on adolescent (aged 10-19 years) use of pornography.

Multimedia Appendix 3 Search strategies used for systematic literature search on adolescent (aged 10-19 years) use of pornography.

Multimedia Appendix 4 Summary, definition of exposure, and grading of quality of studies included in the present systematic review.

## Abbreviations

PRISMA: Preferred Reporting Items for Systematic Reviews and Meta-Analyses
