# Supplementary material for: Exposure to Pornography and Adolescent Sexual Behavior: Systematic Review
Source: J Med Internet Res. 2023 Feb 28;25:e43116. doi: 10.2196/43116 (PMC10015350; doi:10.2196/43116)
Supplement: Multimedia Appendix 3 [file jmir_v25i1e43116_app3.docx]

**Multimedia Appendix 3**

**Search Strategy**

*Medline via Ovid (1946 to present) [n=1438]*

Adolescent Behavior/ OR Adolescent/ OR Adolescent Health/ OR Adolescent Development/ OR adolescen*.mp. OR Child Behavior/ OR Child/ OR Child Development/ OR Child Health/ OR child*.mp. OR teen*.mp. OR Young Adult/ OR young adult*.mp. OR Young people*.mp. OR youth*.mp. OR Students/ OR student*.mp.

AND

erotica/ OR erotic*.mp. OR sexual* explicit*.mp. OR internet porn*.mp. OR porn*.mp.

*PsycINFO via Ovid (1806-present) [n=2089]*

Adolescent Behavior/ OR Adolescent Health/ OR Adolescent Development/ OR adolescen*.mp. OR Child Behavior/ OR Child Health/ OR child*.mp. OR teen*.mp. OR young adult*.mp. OR Young people*.mp. OR youth*.mp. OR Students/ OR student*.mp.

AND

erotic*.mp. OR sexual* explicit*.mp. OR internet porn*.mp. OR pornography/ OR porn*.mp.

*Embase via OvidSP (1947 - present) [n=1766]*

Adolescent Behavior/ OR Adolescent/ OR Adolescent Health/ OR Adolescent Development/ OR adolescen*.mp. OR Child Behavior/ OR Child/ OR Child Development/ OR Child Health/ OR child*.mp. OR teen*.mp. OR Young Adult/ OR young adult*.mp. OR Young people*.mp. OR youth*.mp. OR Students/ OR student*.mp.

AND

erotica/ OR erotic*.mp. OR sexual* explicit*.mp. OR internet porn*.mp. OR pornography/OR porn*.mp.

*Cochrane Composite via Ovid [n=81]*

*Cochrane [EBM Reviews - Cochrane Methodology Register 3rd Quarter 2012, EBM Reviews - Cochrane Database of Systematic Reviews 2005 to November 23, 2022, EBM Reviews - ACP Journal Club 1991 to October 2022, EBM Reviews - Cochrane Clinical Answers November 2022, EBM Reviews - Database of Abstracts of Reviews of Effects 1st Quarter 2016, EBM Reviews - Cochrane Central Register of Controlled Trials October 2022, EBM Reviews - Health Technology Assessment 4th Quarter 2016, EBM Reviews - NHS Economic Evaluation Database 1st Quarter 2016*

adolescen*.mp. OR Adolescent Behavior/ or Adolescent/ or Adolescent Health/ or Adolescent Development/ OR child*.mp. OR Child Behavior/ or Child Development/ or Child/ or Child Health/ OR teen*.mp. OR young adult*.mp.OR Young Adult/ OR Young people*.mp. OR youth*.mp. OR student*.mp. OR Student/

AND

erotic*.mp. OR sexual* explicit*.mp. OR internet porn*.mp. OR porn*.mp.

*Cinahl via EBSCOhost [n=629]*

Adolescent Behavior/ OR Adolescence/ OR Adolescent Health/ OR Adolescent Development/ OR adolescen* OR Child Behavior/ OR Child/ OR Child Development/ OR Child Health/ OR child* OR teen*OR Young Adult/ OR young adult* OR Young people* OR youth* OR Students/ OR student*

AND

erotic* OR sexual* explicit* OR internet porn* OR pornography/ OR porn*

*Web of Science [n=3874]*

Adolescent Behavio*r* OR adolescen* OR Adolescent Health*.mp. OR Adolescent Development*.mp. OR adolescen*.mp. OR Child Behavio*r* OR Child* OR Child Development* OR Child Health* OR child* OR teen* OR young adult* OR Young people* OR youth* OR student*

AND

erotic* OR sexual* explicit* OR internet porn* OR porn*
