# Supplementary material for: Exposure to Pornography and Adolescent Sexual Behavior: Systematic Review
Source: J Med Internet Res. 2023 Feb 28;25:e43116. doi: 10.2196/43116 (PMC10015350; doi:10.2196/43116)
Supplement: Multimedia Appendix 4 [file jmir_v25i1e43116_app4.docx]

**Multimedia Appendix 4: Summary, definition of exposure and grading of quality of studies included in the present review.**

### ***Multimedia Appendix 4.1.* Assessment of cross-sectional studies based on Newcastle Ottawa Scale**

1) * = all subjects in target population OR random sampling. 2) * = Sample size is justified and explained. 3) * = Response rate is given with comparisons between non-respondents and respondents established. 4) ** = exposure is measured by validated measurement tool. * = non-validated measure tool, but is described or available. 5) * = controls for one factor (age, gender etc.) * = controls for any additional factors. 6) * = Independent blind assessment, * = self-report, * = record linkage, 7) * = statistical tests are described clearly and the measurement of the association is presented, including confidence intervals and the probability level (p value).

| Study | Selection (maximum 5 asterisk) | | | | Study adjustments (maximum 2 asterisks) | Outcome (maximum 3 asterisk) | | Overall (maximum 10 stars) |
| --- | --- | --- | --- | --- | --- | --- | --- | --- |
|  | 1. Representative samples | 2. Sample size | 3. Non-respondents | 4. Ascertainment of Exposure | 5. Controlling confounders | 6. Assessment of Outcome | 7. Statistical test |  |
| Atwood 2012 [32] | * |  | * | * | * | * | * | 6 |
| Bukenya 2020 [34] | * | * |  | * | ** | * | * | 7 |
| Barter 2021 [33] | * | * |  | * | ** | * | * | 7 |
| Donevan 2017 [35] | * | * |  | ** | ** | * | * | 8 |
| Kim 2016 [36] | * | * | * | * | * | * | * | 7 |
| Nelson 2019 [37] | * |  |  | ** | ** | * | * | 7 |
| Ng 2016 [38] | * | * | * | * | ** | * | * | 8 |
| Rostad 2019 [39] | * |  |  | ** | ** | * | * | 7 |
| Sahay 2013 [43] | * |  | * | * | * | * | * | 6 |
| Ševčíková 2014 [40] | * | * |  | ** | ** | * | * | 8 |
| Sit-Haidah 2017 [44] | * |  |  | * | ** | * | * | 6 |
| Svedin 2011 [41] | * | * |  | * | ** | * | * | 7 |
| Wright 2020 [42] | * |  |  | * |  | * |  | 3 |

### ***Multimedia Appendix 4.2.* Assessment of longitudinal studies based on Newcastle Ottawa Scale**

1) * = all subjects in target population OR random sampling. 2) * = Non-exposed cohort drawn from same community as exposed. 3) ** = validated scale of exposure, * = structured interview/self-report. 4) * = controls for one factor (age, gender etc.) * = controls for any additional factors. 5) * = Independent blind assessment/self-report/record linkage. 6) * = complete follow up/follow-up rate of 80-100%. 7)* = outcome is present as baseline at lower levels.

| Study identifier | Selection (4 asterisk) | | | Comparability (2 asterisk) |  | | | Total |
| --- | --- | --- | --- | --- | --- | --- | --- | --- |
|  | 1. Representative samples | 2. Non-exposed cohort | 3. Ascertainment of Exposure | 4. Controlling confounders | 5. Ascertainment of outcome | 6. Follow-up cohort | 7. Outcome at baseline |  |
| Averdijk 2020 [45] | * | * | * | * | * |  |  | 5 |
| Koletić 2019 [46] | * | * | * | ** | * |  | * | 7 |
| Lin 2020 [47] | * | * | * | ** | * |  |  | 6 |
| Matković 2018 [48] | * | * | * | ** | * |  | * | 7 |
| Peter 2011 [49] | * | * | ** | ** | * | * |  | 8 |
| Vandenbosch 2013 [50] | * | * | ** | ** | * |  | * | 8 |

### ***Multimedia Appendix 4.3.* Summary of details of the studies included in systematic review.**

| **Author** | **Location of study** | **Sample size. (n =, % F/M/NB, gender or F/M sex as reported)** | **Age of sample, M (SD)** | **Type of study** | **Sampling strategy** | **Description of sample** | **Risk of Bias Assessment** | **Data collection method** | **Relevant outcome assessed** | **Control variables as reported by study** |
| --- | --- | --- | --- | --- | --- | --- | --- | --- | --- | --- |
| Atwood^a^ (2012) [32] | Bangkok, Thailand | 420 (50.5% Female) | 13.45 (0.5) | Cross sectional | Random and probability to proportionate sampling to be representative of Bangkok | 91.2% ethnic Thai, adolescents living in households with parents. | Moderate | audio-computer assisted self-interviews | Having ever had sex (referred to as “going all the way”, or “doing it”). | parental disapproval  grades  having a boyfriend/ girlfriend  sexual refusal self efficacy |
| Averdijk^a^ (2020) [45] | Zurich, Switzerland | 1197 (50.3% Male)  Original population, 1675 (52% Male) | 13.7 (0.37) at baseline  17.4 (0.37) at final wave | Longitudinal (analysed 4 years, 3 waves) | Stratified random sampling of school students based on school size and socio-economic status. | School children, 48% of parents had adolescents of non-swiss origin, and average level of education (mean 2, SD = 0.78 of 1 being academic high school and 4 being special needs). | Moderate | Classroom-based paper survey | Sold or bought sex | Gender |
| Barter^a^ (2021) [33] | England, Italy, Norway, Bulgaria and Cyprus. | 4564 (NR, Total adolescents who responded to outcome sexual aggression indicative of forced sex, n=1677 (male), n=1515 (female).  Analysis conducted on, n=3277) | Overall M(SD) NR, 14.7 in England, 15.3 in Italy, 14-17 | Cross-sectional | Data were from a randomly selected number of schools (45 across examined countries) in each country, where approximately 1000 participants in each country except Cyprus where the number was 500. | Adolescents aged 14 to 17 who reported having every experienced at least one relationship | Low | Paper surveys | Sexual aggression (including forced sex) | violence in the household and negative gender attitudes, age and country of residence, Bullying, aggressiveness of friends, |
| Bukenya^a^ (2020) [34] | Iganga-Mayuge Health and Demographic Surveillance Site, Eastern Uganda | 598 (52% male) | 14.2 (2.6) | Cross sectional | Random selection of 600 adolescents from surveillance site. | 12.7% of adolescents in sample did not go to school, residing in households. | Low | Face to face questionnaires | Having ever had sex (defined as vaginal, anal or oral) | age, education level, parental relationship, substance use, sexting experience, history of being bullied, experienced physical arrack, intentions of participating in verbal sex jokes and engagement in labour activities |
| Donevan^a^ (2017) [35] | Medium and large sizes Towns, Sweden | 730 (49.2% Female)  Analysed only males (n=371) | ND, Analysed only 18-year-olds | Cross sectional | All available students from select towns (77% of 946 eligible responded) | 18-year-old adolescents attending senior high school. | Low | Paper based surveys at classroom | First age of sex (defined as vaginal, anal, or oral) | NA |
| Kim^a^  (2016) [36] | South Korea | 2387 (74.6% Male) | ND, 12-19 age range | Cross sectional | Secondary data analysis of KYRBWS-VIII, nationally conducted, random selection of one class from nationally representative sample frame. | 12–19-year-old adolescents having reported to have had sex, 16.7% of sample have low family affluence score. | Low | anonymous self-report questionnaire at school computer labs. | Sexually transmitted infections | Gender  Weekly allowance  Family affluence  Living with family  stress, suicidal thoughts, vigorous physical activity  drug experience  internet use outside time  age at first intercourse  method of contraception in most recent intercourse |
| Koletić^a^ (2019) [46] | Croatia (cities of Zagreb and Rijeka) | Zagreb cohort: n=1057 (35.6% Male), M=16.14 (0.45) at baseline eligible for analysis in study.    Rijeka cohort:  n=1071 (38.4% Male) M=15.82 (0.49) at baseline eligible for analysis in study  Older students (OR=1.97, p=0.045) and males (OR=1.48, p=0.034) were likely to drop out of study. | At baseline,  Rijeka: 15.82 (0.49)  Zagreb: 16.14 (0.54) | Longitudinal (2.5 years, 6 waves in Rijeka, 5 waves in Zagreb) | Purposive sample of select schools invited to participate. | High school adolescents, approximately one third of parents college educated | Low | Online and paper-based questionnaires | Sex with multiple partners and condomless sex | Sensation seeking  Pubertal timing  Parent education level |
| Lin^a^ (2020) [47] | Taiwan | N = 2690 at baseline (51% male). Included in analysis:  Early sexual debut and condomless sex, n = 2054, and multiple partner, n = 1477 | At baseline,  13.3 (0.49) and at final wave for analysis, 24.3 (0.47) | Longitudinal (11 years, 10 waves) | Randomly selected 7^th^ graded class from select schools (unclear on how these schools were selected). | High school students, approximately 30-40% of parents below college education. | Moderate | Online and classroom-based questionnaire | Early sexual debut (threshold of 17), condomless sex and multiple sexual partners | gender, parents' education, monthly income, family intactness, number of siblings, parental control, family cohesion, class rank at wave 1, health status, depressive symptoms, dating experience  School fixed effects (reported school of the particpant) |
| Matković^a^ (2018) [48] | Rijeka, Croatia | n=1037 at baseline (NR).  Included in analysis for outcome: n=866 (39% Male) adolescents who reported no sexual experience at baseline and participated in at least two consecutive waves  Replication analysis conducted in Zagreb panel, n=793 adolescents  At baseline, 16.1 (0.44), and 67.8% Female | At baseline, mean age is 15.8 (0.50)  Replication analysis in panel (At baseline, 16.1 (0.44), and 67.8% Female) | Longitudinal (16 months, three waves from baseline, 4 months apart) | Selected all of 14 larger secondary schools in Rijeka (7 small schools less than 50 excluded), 63% of the city’s adolescent population at this particular high-school grade level. | High school students, non-respondents were more likely to attend a vocational or technical school than prestigious (b = .98, p < .05, and b = .50, p < .05) | Low | Self-administered paper and pencil survey in a classroom setting, with portable screens for confidentiality. | Early age of first sex (included in review as study examined a population at baseline, whose mean age was <16) | pubertal status, school type, and age at first exposure to SEM, age, sensation seeking, parental monitoring, and a contextual variable indicative of peer participation in sex. |
| Nelson^a^  (2019) [37] | USA | 206 (Male only) | 16 (1.0) | Cross sectional | Online sample recruited based on eligibility criteria | Cis-gender male adolescents self-identified as gay/bisexual, residing in US and have personal address. 51% racial minority, 95% in school. | Low | Online surveys via RedCap | Condomless anal sex | age, race/ethnicity, gender (as sample includes one gender) |
| Ng^b^  (2016) [38] | Singapore | 300 (Male only) | Median = 8  IQR = 18-19 | Cross sectional | Baseline assessment of adolescents enrolled into a clinical trial for sexual health intervention. 73% of those eligible in study consented to participate. | Heterosexual adolescents (defined as having sex with female), unmarried and visiting the clinic for the first time. This STI clinic is representative of the core group of STI transmission at Singapore. | Low | Face to face interviews followed by self-administered questionnaires. | Bought sex with female sex worker | Adjusted for alcohol consumption, rebellious attitudes, self-esteem, perceived external control, academic performance, participation in co-curricular activities, age of first sex and history of having a sexually active girlfriend |
| Peter^a^ (2011) [49] | Netherlands | 1,445 adolescents included for analysis (51% male) | At base line, 14.49 (SD=1.68) | Longitudinal, | National representative, random selection of a pool of adolescents and adults. | 12-19 adolescents, general population, 95% heterosexual. | Low | online questionnaire | Condomless sex | Controlled for sensation seeking, life satisfaction, peer relationships, sexual orientation, relationship status, number of lifetime sex partners, and casual condomless sexual behavior of friends |
| Rostad^a^  (2019) [39] | Rhode Island and Massachussets in USA | 1694 (54.5% Female) | 15.42 (0.65) | Cross sectional | Data were from a baseline survey administered in a randomized clinical trial of a school-based sexual assault prevention program for high school youth.  All grade 10 students from 27 selected schools who were eligible and consented | Adolescents in grade 10 of select high schools who reported having a dating relationship in the past year. 31% Racial minority and identified exclusively as male or female. | Low | Surveys on provided laptops or pen and paper, self-administered. | Sexual aggression (including forced sex) | Age, history of suspension or expulsion, heavy drinking, marijuana use, rape myth acceptance, gender equitable attitudes  Gender stratified analysis |
| Sahay^a^  (2013) [43] | Pune, Maharshasta, India | 205 (47% female) | 14.6 (NR) | Case-control | School selected based on parental consent at interactive workshops. Selected all consenting 9^th^ and 11^th^ grades students. 205 of 910 eligible selected based on case-control analysis. | Case control analysis. Case defined as adolescents who had sex, and control of those who did not have sex selected in ratio of 1:4 (case: control), accounting for sex, urban or rural locale, and type of schools. | Moderate | self-administered structured questionaiire at school classrooms | Having had sex (mean age of population below 16) | Medium of instruction (english/native vernacular), history of accessing reproductive and sexual health material, history of sexual abuse, self-reported relationship with parents, report STD symptoms |
| Ševčíková^a^  (2014) [40] | Austria, Belgium, Bulgaria, Cyprus, the Czech Republic, Denmark, Estonia, Finland, France, Greece,  Hungary, Ireland, Lithuania, the Netherlands, Norway,  Poland, Portugal, Romania, Slovenia, Spain, Sweden,  Turkey and the UK) | 11,712 (50% female) | Overall NR  No exposure to porn:  13.24 (1.66)  Intentional exposure to porn:  14.54 (1.40)  Unintentional exposure to porn:  14.12 (1.57) | Cross sectional | 1000 participants randomly selected from each country based on random probability sampling. For study, included participants who responded to all target questions (about porn exposure and sexual behaviours). | Nationally representative, adolescents in families aged 9-16. | Low | face to face interviewer administered and self-completion paper based or PC based. | Having had sex (mean age of population below 16) | Age, gender, sensation seeking, private use, time online daily, digital, active or restrictive parental mediation, liberalism of country and interaction terms (gender liberalism, parental restriction/mediation and liberalism) |
| Siti-Haidah^c^  (2017) [44] | Kuala Lumpur City, Malaysia | 215 (Female only) | 12-19 (NR, 75% above 15) | Case-control | Case of pregnant adolescents recruited from government shelters. Control of non-pregnant adolescents purposively selected to represent case by age and gender. | Case-control analysis. Case of pregnant adolescents in government shelters, and control of school students aged 12-19 (75% aged 15 and over). Low parental education in sample (>50%) and low monthly income (>50%) for pregnant adolescents. | Moderate | Questionnaires (Medium NR, assuming in person paper, as states give questionnaire) | Teenage pregnancy | race, family income and parents’ education level |
| Svedin^a^  (2011) [41] | Stockholm, Malmo, Lulea, Haparanda and Falkoping (mix of city, large and small), and included students in surrounding areas, Sweden | 4377 (NR)  18.15 (0.74) years  Analysed sample:  N=172-200 (100% Male) adolescents who were frequently exposed to porn  N = 1429-1702 (100% Male) reference group of male adolescents who were less frequently exposed to porn | 18.15 (0.74) | Cross sectional | Proportional sampling of schools to be selected in Sweden. Final number consented to participate and eligible due to complete surveys (response rate of 77.2%). Minimal number of females reported frequent exposure to porn thus excluded females from analyses in study. | Senior high school male adolescents, nationally representative of Sweden socio-economically. | Low | Paper based questionnaires in classroom setting | Early age of first sex  Sexually coercive behaviour (penetration)  Bought sex and sold sex | Immigrant status, status of living with parents, living in large city, parental care cut offs, alcohol use, drug use, health score, history of having stolen something, reprimand by headmaster, argument with teacher, sexual intention |
| Vandenbosch^a^ (2013) [50] | Flanders, Belgium | 639 (58% male) | At baseline, 14.78 (1.18) | Longitudinal (6 months) | 11 schools selected from all schools willing to participate in Flanders (unclear on school selection) | High school students, 65.7% attending general education program intended for tertiary study preparation | Low | Paper-based questionnaire in classroom | Sexual intercourse (mean age of examined below 16) | country of origin,  gender, age, educational level, communication with  parents, communication with peers and sensation  seeking |
| Wright^a^  (2020) [42] | US, nationally representative | N = 95, (54% female) | NR.  For those who engaged in condom less sex, 45.4% was aged 18 | Cross sectional | Data from the National  Survey of Porn Use, Relationships, and Sexual Socialization (NSPRSS).  Population based, probability sample of 14-60. Analysed sub-sample of n = 614 | Adolescents from nationally representative sample. N = 95 of 614 engaged in condomless sex (89.2% heterosexual, 53.1% white, non-hispanic) | High | Online surveys and panel | Condomless sex | Parental communication about sex was used as a conditional variable. |

ND = Not determined by original study.

^a^Study examined gender (F/M/NB)

^b^Examined male adolescents, no clear reference to gender/sex.

^c^Examined case-control analysis including pregnant and non-pregnant female adolescents, no clear reference to gender/sex.

***Multimedia Appendix 4.4.* Definitions of pornography used in each study.**

| **Author** | **Definition of porn used as exposure** | **Recall period** | **Medium of porn assessed** | **Assessment of Frequency** | **Type of porn assessed** | **Scale used to assess the exposure** |
| --- | --- | --- | --- | --- | --- | --- |
| Atwood (2012) [32] | Not stated in methods | Ever | Internet Websites and television | Not assessed | Not defined, general. | Created for study  Example item provided. |
| Averdijk (2020) [45] | Not stated in methods | Not described in methods, assessed by frequency. | Adult films and any internet content | Assessed Frequency, 7 point scale ranging from never to daily. | Not defined, general. | Created for study.  All items described |
| Barter  (2021) [33] | Not stated in methods | Unclear, asked if participant regularly watch pornography | Online, otherwise unstated. | Suggested, unclear in what constitutes as regular exposure | Not defined, general | Created for study.  All items described |
| Bukenya (2020) [34] | Phrased as “Sexually explicit content” | Ever | Films and images | Not assessed | Not defined, general. | Adapted from QS from global school-based health survey |
| Donevan (2017) [35] | Not stated in methods | Ever | Not stated or described in methods | Assessed Frequency.  Frequent use is defined as daily exposure to pornography. Average use is defined as weekly use. | Examined various categories of porn exposure (e.g., softcore, hard, etc.) | Created for study.  All items described |
| Kim (2016) [36] | Defined as preference for adult content, aged 19 or over | Not described in methods | Internet Websites | Not assessed | Not defined, general preference. | Created for study, single item described. |
| Koletić (2019) [46] | Defined as any material which openly depicts sexual activity (uncensored). | Last 6 months | Not defined | Assessed Frequency, 8 point scale ranging from never to several times a day. | Not defined, general. | Single item commonly acknowledged (Bergkvist & Rossiter, 2007; Diamantopoulos et al., 2012). Item described |
| Lin (2020) [47] | Defined as adult only or restricted (R-rated) media, which is equivalent to “sexually explicit material” in Mandarin. | Ever | Internet websites, magazines, comic books, novels, films, and other. | Not assessed | Not defined, general. | Created for study, single item described. |
| Matković (2018) [48] | Defined for participants, stated “any material which openly depicts sexual intercourse or other sexual acts. Material which shows naked bodies but not sexual intercourse or other sexual activity does not belong to pornography as here defined.” | Past 6 months | Not defined | Assessed.  Low use category defined as pornography use once a month or less  High use category defined as pornography use daily | Not defined, general | Created for study, single item described. |
| Nelson (2019) [37] | Phrased as “sexually explicit media” | Ever | Internet websites, on phones and magazines | Assessed Frequency  A 9-point scale ranging from more than once an hour to less than once a month.  Also assessed how long participant viewed porn per session, ranging from less than a minute to 61 minute) | Analysed viewing condom-less anal sex in porn | Adapted from previous study in 2014, and 2016 |
| Ng (2016) [38] | Porn not defined in methods. Stated as “banned media in Singapore i.e., pornography) | Not described in methods, assessed by frequency. | Not stated in methods. | Assessed Frequency.  4-point scale ranging from hardly to almost every time. | Not defined, general | Created for study, single item described |
| Peter (2011) [49] | Defined as material with clearly exposed genitals (to participants) | Last 6 months | Online or downloaded pictures, and videos | Assessed Frequency.  7-point scale ranging from never to several times a day | Not defined, general | Created for study, multiple items described |
| Rostad (2019) [39] | Defined as material depicting a female or females being forced to engage in sexual acts. | Ever | Magazines, videos, films or written books | Not assessed | Violent porn defined as material depicting a female or females being forced to engage in sexual acts. | Three items from the Social Norms Measure (Boeringer, Shehan, & Akers, 1991) |
| Sahay (2013) [43] | Porn itself not defined in methods. | Not described in methods. | Albums, movies or internet websites. | Not assessed | Not defined, general. | Created for study, single item described. |
| Ševčíková (2014) [40] | Phrased as “exposure to online sexual material”. | Past year | Internet websites and pop-up images.  Medium used to define intention. Unintentional exposure defined as porn via pop-up ad images | Not assessed | Not defined, general | Created for study, All items described |
|  |  |  | Intentional exposure defined as porn via adult websites. |  |  |  |
| Siti-Haidah (2017) [44] | Porn not defined in methods. | Ever | Pictures, videos on mobile phone.  Books and magazines. | Not assessed | Not defined, general | Created for study,  All items described |
| Svedin (2011) [41] | Porn not defined in methods | Ever | Internet websites, magazines, films. | Assessed Frequency,  Ranging from once to more or less daily. | Not defined, general | Compilation from previously validated surveys, all items described |
| Vandenbosch (2013) [50] | Defined as pictures or videos with exposed genitals or people having sex. | Last 6 months | Internet websites (referring to videos and pictures) | Assessed frequency, 3-point scale ranging from non-users to several times per day. | Not defined, general. | Created for study based on Peter & Valkenburg 2008, example items described |
| Wright (2020) [42] | sexually explicit pictures, videos, or  livestreams showing clearly exposed genitals, or, in which  people are clearly shown having sex, such as oral sex, vaginal sex, or anal sex | Ever | Internet websites, videos and pictures | Not assessed | Examined various categories of porn (for e.g., amateur, facial ejaculation etc.) | Created for study, all items described. |
